# *Pneumocystis murina* promotes inflammasome formation and NETosis during *Pneumocystis* pneumonia

**DOI:** 10.1128/mbio.01409-24

**Published:** 2024-07-02

**Authors:** Steven G. Sayson, Alan Ashbaugh, Aleksey Porollo, George Smulian, Melanie T. Cushion

**Affiliations:** 1 Department of Internal Medicine, University of Cincinnati College of Medicine, Cincinnati, Ohio, USA; 2 The Veterans Affairs Medical Center, Cincinnati, Ohio, USA; 3 Division of Human Genetics, Center for Autoimmune Genomics and Etiology, Cincinnati Children’s Hospital Medical Center, Cincinnati, Ohio, USA; 4 Division of Biomedical Informatics, Cincinnati Children’s Hospital Medical Center, Cincinnati, Ohio, USA; 5 Department of Pediatrics, University of Cincinnati, Cincinnati, Ohio, USA; University of Minnesota Medical School, Minneapolis, Minnesota, USA; Tulane University School of Medicine, New Orleans, Louisiana, USA

**Keywords:** *Pneumocystis*, infectious disease, neutrophils, opportunistic fungi, immunity, *Pneumocystis pneumonia*, *Pneumocystis murina*, NETosis, inflammation

## Abstract

*Pneumocystis jirovecii* pneumonia (PjP) poses a serious risk to individuals with compromised immune systems, such as individuals with HIV/AIDS or undergoing immunosuppressive therapies for cancer or solid organ transplants. Severe PjP triggers excessive lung inflammation, resulting in lung function decline and consequential alveolar damage, potentially culminating in acute respiratory distress syndrome. Non-HIV patients face a 30%–60% mortality rate, emphasizing the need for a deeper understanding of inflammatory responses in PjP. Prior research emphasized macrophages in *Pneumocystis* infections, neglecting neutrophils’ role in tissue damage. Consequently, the overemphasis on macrophages led to an incomplete understanding of the role of neutrophils and inflammatory responses. In the current investigation, our RNAseq studies on a murine surrogate model of PjP revealed heightened activation of the NLRP3 inflammasome and NETosis cell death pathways in their lungs. Immunofluorescence staining confirmed neutrophil extracellular trap (NET) presence in the lungs of the *P. murina*-infected mice, validating our findings. Moreover, isolated neutrophils exhibited NETosis when directly stimulated with *P. murina*. Isolated NETs compromised *P. murina* viability *in vitro*, highlighting the potential role of neutrophils in controlling fungal growth and promoting inflammation during *P. murina* pneumonia through NLRP3 inflammasome assembly and NETosis. These pathways, essential for inflammation and pathogen elimination, bear the risk of uncontrolled activation leading to excessive tissue damage and persistent inflammation. This pioneering study is the first to identify the formation of NETs and inflammasomes during *Pneumocystis* infection, paving the way for comprehensive investigations into treatments aimed at mitigating lung damage and augmenting survival rates for individuals with *PjP*.

**IMPORTANCE:**

*Pneumocystis jirovecii* pneumonia (PjP) affects individuals with weakened immunity, such as HIV/AIDS, cancer, and organ transplant patients. Severe PjP triggers lung inflammation, impairing function and potentially causing acute respiratory distress syndrome. Non-HIV individuals face a 30%–60% mortality rate, underscoring the need for deeper insight into PjP’s inflammatory responses. Past research focused on macrophages in managing *Pneumocystis* infection and its inflammation, while the role of neutrophils was generally overlooked. In contrast, our findings in *P. murina*-infected mouse lungs showed neutrophil involvement during inflammation and increased expression of NLRP3 inflammasome and NETosis pathways. Detection of neutrophil extracellular traps further indicated their involvement in the inflammatory process. Although beneficial in combating infection, unregulated neutrophil activation poses a potential threat to lung tissues. Understanding the behavior of neutrophils in *Pneumocystis* infections is crucial for controlling detrimental reactions and formulating treatments to reduce lung damage, ultimately improving the survival rates of individuals with PjP.

## INTRODUCTION

*Pneumocystis jirovecii* pneumonia (PjP) is a leading cause of mortality in hospitalized individuals with HIV/AIDS. However, the incidence of PjP has increased in cancer patients and individuals who have received organ transplants requiring immune-suppressing treatments. Among all hospitalizations for PjP, malignancy stands as the most prevalent predisposing factor, accounting for 46.0%–55.7% of cases, followed by HIV at 17.8% (1–4). Within immunocompetent hosts, signaling cascades initiated by CD4+ T cells and B cells efficiently trigger *P. jirovecii* clearance with minimal inflammation (5). Conversely, immunosuppressed hosts experience an influx of various immune cells into their lungs, including T lymphocytes, polymorphonuclear neutrophils, and other leukocytes (6–9). This can lead to a profound inflammatory response which leads to considerable morbidity and mortality. Severe PjP is characterized by a neutrophilic inflammatory response presenting with decreased pulmonary function, alveolar damage, and respiratory failure (10, 11). Furthermore, bronchial alveolar lavage (BAL) neutrophilia has been shown to be a predictor of poor prognosis and increased mortality in PjP (12).

Previous research has primarily centered on macrophage polarization and its role in clearance of *Pneumocystis* from infected hosts. Both classically activated M1 and alternatively activated M2 macrophages are involved in *Pneumocystis* clearance. Immunocompetent mice show preferential activation of the M2 phenotype during *P. murina* exposure, while immunosuppressed hosts show enhanced M1 polarization (13). M1-polarized macrophages are effective fungicidal cells and produce a substantial cytokine and chemokine response (14). These secretions not only eliminate infectious organisms but also signal for further recruitment of immune cells.

Pattern recognition receptors, such as C-type lectin receptors dectin-1/2 and toll-like receptors (TLRs), expressed by innate immune cells bind to pathogen-associated molecular patterns and damage-associated molecular patterns (15). Dectin-1/2 and TLR2 have been identified as important receptors in antigen recognition to *P. murina* in mice, leading to immune responses such as pro-inflammatory cytokine release and fungal clearance (16–18).

Neutrophils play a crucial role in host defenses against pathogenic organisms. During *Candida albicans* and *Aspergillus fumigatus* infections in mice, neutrophils aid in controlling fungal growth by phagocytosis and reactive oxygen species (ROS) generation for effective pathogen killing (19, 20). Beyond phagocytosis and ROS generation, neutrophils employ additional antimicrobial activity, including degranulation, cytokine production, and NETosis. NETosis results in the expelling of DNA to form neutrophil extracellular traps (NETs) that ensnare and kill pathogens (21, 22). These NETs are decorated with various proteins, such as histones, neutrophil elastase (NE), calprotectin, myeloperoxidase (MPO), and other antimicrobial proteins (23, 24).

PjP is associated with an accumulation of neutrophils in the lungs (8, 9). Additionally, levels of interleukin-8, a chemotactic and activating agent for neutrophils, within BAL fluid are directly associated with the clinical severity of pneumonia and serve as a prognostic marker for mortality risk and the likelihood of significant respiratory compromise (25). A previous study by Swain et al. found that neutrophils and reactive oxygen species do not contribute to pulmonary tissue damage nor play a major role in clearance of *Pneumocystis* (26). Consequently, despite the significant influx of neutrophils into the lungs during *Pneumocystis* infection, their role in inflammation has been both overlooked and understudied, resulting in a significant knowledge gap.

This study revealed an up-regulation in the expression of genes associated with the NLRP3 inflammasome and NETosis in the lungs of mice infected with *P. murina*. These processes are essential for the elimination of pathogens from the host system. However, overactivation of these pathways leads to heightened inflammation and tissue damage, emphasizing the need for a thorough understanding of their regulation in the context of *Pneumocystis* infection. This investigation intended to elucidate the roles of neutrophil populations during PjP to establish the groundwork for therapeutic strategies aimed at mitigating excessive inflammatory responses and alveolar damage.

## RESULTS

### Signaling pathways involved with inflammatory processes increase during *P. murina* infection

**Fig 1:**
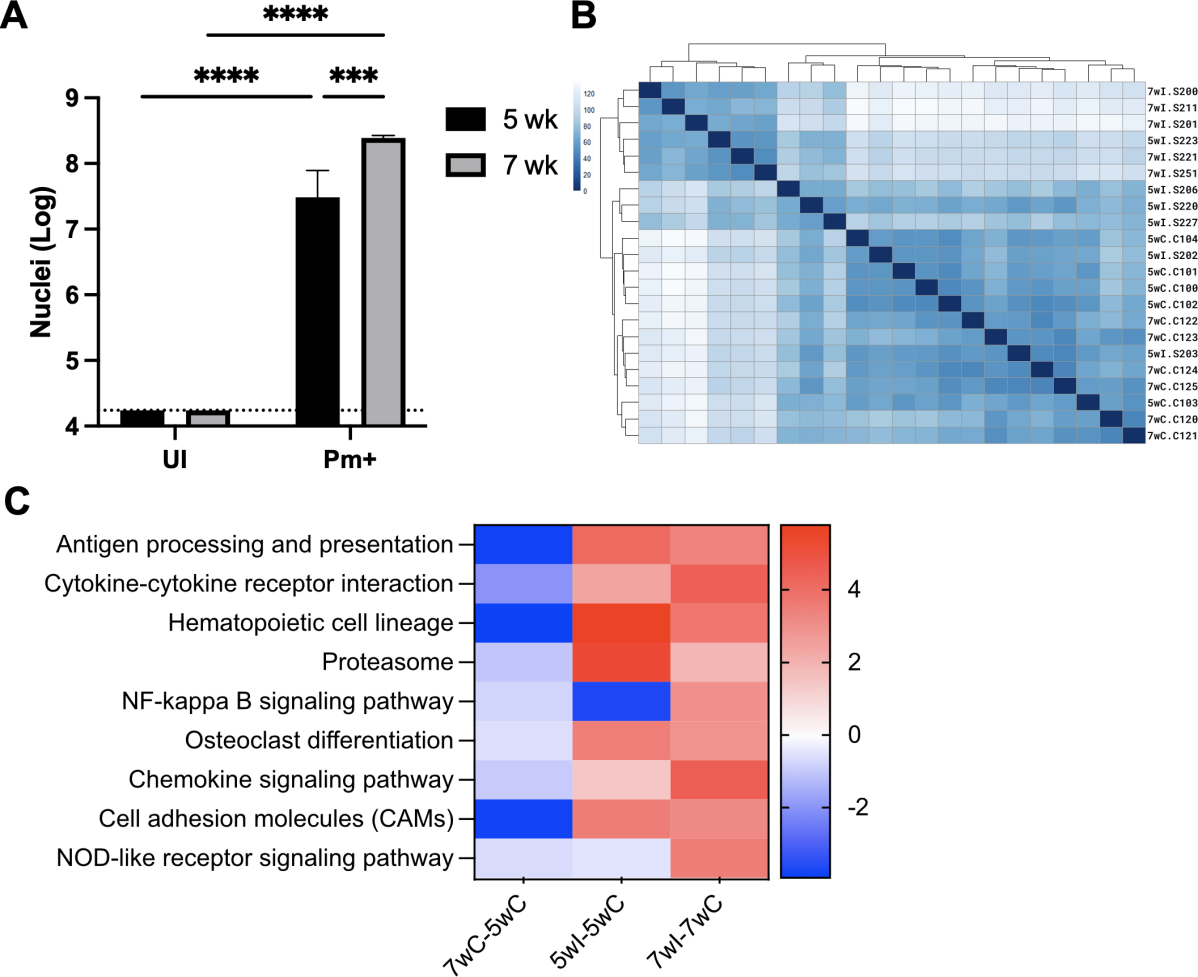
Signaling pathways associated with inflammatory processes are up-regulated as *Pneumocystis murina* infection progressed in mice. Immunosuppressed mice were exposed to uninfected or previously *P. murina*-infected mice. (**A**) After 5 or 7 weeks of exposure, the fungal burden in the lungs was enumerated. Dashed line, limit of microscopic enumeration. ***, *P* < 0.001. ****, *P* < 0.0001. (**B**) Heatmap of sample-to-sample distances. 5wC and 7wC uninfected samples cluster near the bottom, and 5wI clusters in the middle, while 7wI clusters at the top of the heatmap. (**C**) Heatmap comparison analysis of gene set enrichment analysis on KEGG pathways relating to signaling. Data are represented as logFC.

To gain a better understanding of the changes in host immune response during the development of *P. murina* infection in mice, lung samples were analyzed from both uninfected and *P. murina*-infected immunosuppressed mice at 5- and 7-week intervals after initial exposure to *P. murina*. Mice with a 7-week infection exhibited a higher fungal burden than those with 5-week infections (Fig. 1A). Differential gene expression analysis revealed that gene expression patterns across different samples were comparable between biological groups (Fig. 1B). Control samples from both the 5-week (5wC) and 7-week (7wC) groups clustered at the bottom, while infected samples from the 5-week (5wI) group clustered in the middle, and those from the 7-week (7wI) group clustered at the top of the heatmap. A gene set enrichment analysis indicated a significant increase in signaling pathways associated with inflammatory processes as *P. murina* infection progressed in mice (Fig. 1C; Table 1).

**TABLE 1:** Functional gene set enrichment analyses^*b*^

Pathway	KEGG ID	NumGenes^*a*^	*P*-value	FDR
Antigen processing and presentation	mmu04612	77/91	1.37E−17	3.25E−16
Cytokine-cytokine receptor interaction	mmu04060	222/270	3.21E−23	3.06E−21
Hematopoietic cell lineage	mmu04640	83/95	2.26E−12	2.81E−11
Proteasome	mmu03050	45/45	9.61E−11	9.82E−10
NF-kappa B signaling pathway	mmu04064	89/103	2.38E−15	4.53E−14
Osteoclast differentiation	mmu04380	116/130	1.45E−10	1.38E−09
Chemokine signaling pathway	mmu04062	162/192	2.30E−08	1.68E−07
Cell adhesion molecules	mmu04514	140/169	4.80E−15	8.57E−14
NOD-like receptor signaling pathway	mmu04621	152/169	1.54E−08	1.22E−07

^*a*^ NumGenes, number genes mapped to the total number of genes in the set.

^*b*^ Enriched pathways based on differential expression.

### NOD-like signaling and NETosis pathways are increased during *P. murina* infection

**Fig 2:**
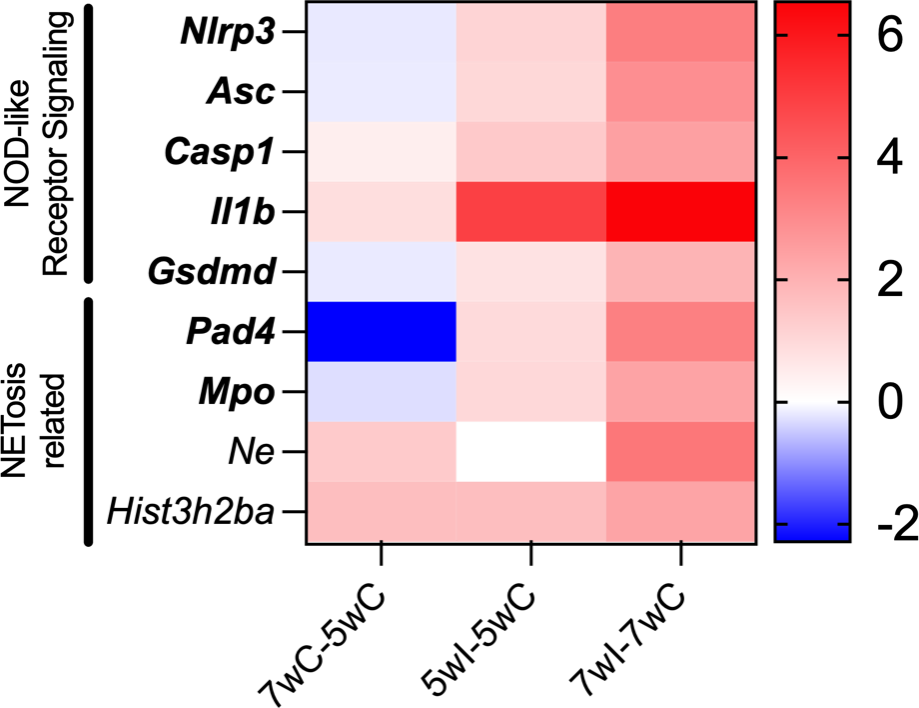
NOD-like receptor signaling and NETosis-related transcripts are up-regulated as *P. murina* infection progressed in mice. Data are represented as log2 fold change between the displayed groups. Data are represented as logFC. Bold, FDR-adjusted *P* < 0.05.

Many of the inflammatory signaling pathways during *Pneumocystis* infections have been extensively studied, such as antigen processing and presentation, cytokine-cytokine receptor interaction, hematopoietic cell lineage, NF-kappa B signaling, chemokine signaling pathway, and cell adhesion molecules (27–31). For that reason, we chose to focus on the understudied NOD-like receptor signaling pathway. In the lungs of mice with both 5- and 7-week infections, there was an observed increase in the expression of key genes associated with the NOD-like receptor signaling pathway that were not present in non-infected mice. These genes included NOD-like protein receptor 3 (*Nlrp3*), apoptosis-associated speck-like protein containing a CARD (*Asc/Pycard*), and caspase 1 (*Casp1*) (Fig. 2). These transcripts encode proteins that form the NLRP3 inflammasome complex, wherein NLRP3 binds to the scaffold protein ASC, which in turn binds to CASP1. The proteolytic enzyme CASP1 component of the NLRP3 inflammasome complex cleaves the pro-inflammatory cytokine interleukin 1 beta (IL-1β) into its mature form. Additionally, CASP1 also cleaves gasdermin D (GSDMD), which inserts into the plasma membrane to facilitate the release of IL-1β to further propagate a proinflammatory response and pyroptotic cell death (32).

The mRNA expression of both *Il-1β* and *Gsdmd* increased in the lungs of mice with 5-week and 7-week infections (Fig. 2). While proinflammatory cytokine IL-1 is a crucial mediator for host resistance and immune cell recruitment during *P. murina* infection (33), inflammasome assembly details have yet been described. Additionally, increased peptidylarginine deiminase 4 (*Pad4*) expression was detected in *P. murina*-infected mice. PAD4 plays a role in chromatin decondensation and expulsion of NETs in a regulated cell death pathway called NETosis (34). Furthermore, PAD4 stimulates NLRP3-inflammasome formation, leading to prolonged inflammation through a positive feedback loop by driving the production of pro-inflammatory cytokines and chemokines (35). This is supported by similar findings that bronchial cells exposed to NETs demonstrated increased secretion of pro-inflammatory cytokine IL-1β (36).

### NET formation in *P. murina*-infected lung tissue

**Fig 3:**
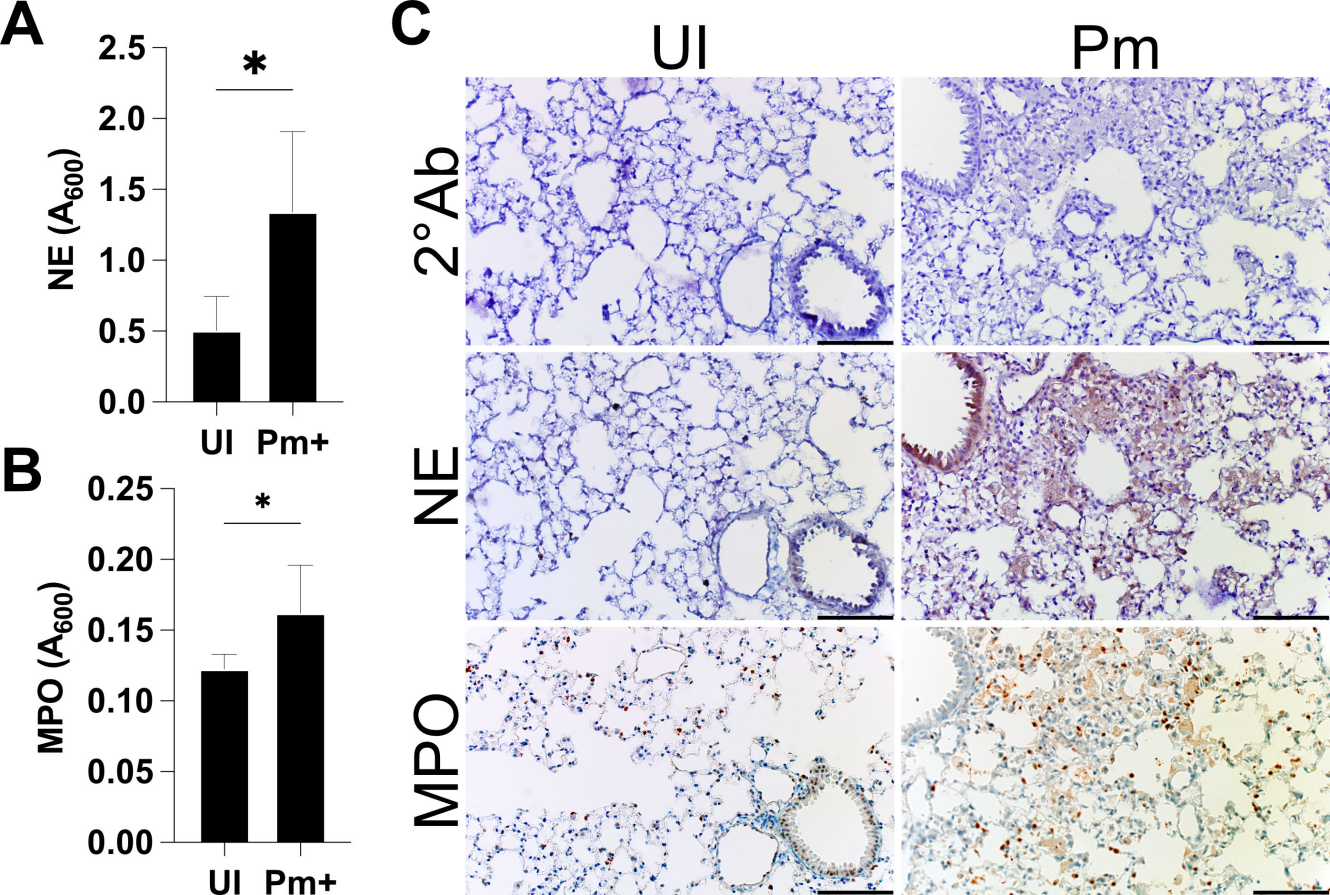
NETosis-related proteins are present in the lungs of *Pneumocystis murina*-infected mice. (**A**) ELISA reveals increased expression of NET components, NE and (**B**) MPO, in the lung tissue from *P. murina*-infected (Pm+) mice over uninfected (UI) mice. *t-test, *P* < 0.05. (**C**) Immunohistochemistry of the lungs of *P. murina*-infected mice. The NETosis markers, NE and MPO, are expressed throughout the thickened alveolar spaces of Pm+ mice. Scale, 100 µm.

Given the increased expression of genes related to NOD-like receptor signaling and the NETosis pathway through differential expression analysis, we sought to confirm the presence of NETs during *Pneumocystis murina* pneumonia (PmP). Increased expression of NE and MPO, which decorate the extracellular DNA in NETs, is significantly increased in the lungs of *P. murina*-infected mice (Fig. 3A and B). Immunohistochemistry analysis revealed expression of NE and MPO, suggesting the presence of NETs within *P. murina*-infected lung tissue (Fig. 3C).

### *P. murina* stimulates NETosis in neutrophils *in vitro*

**Fig 4:**
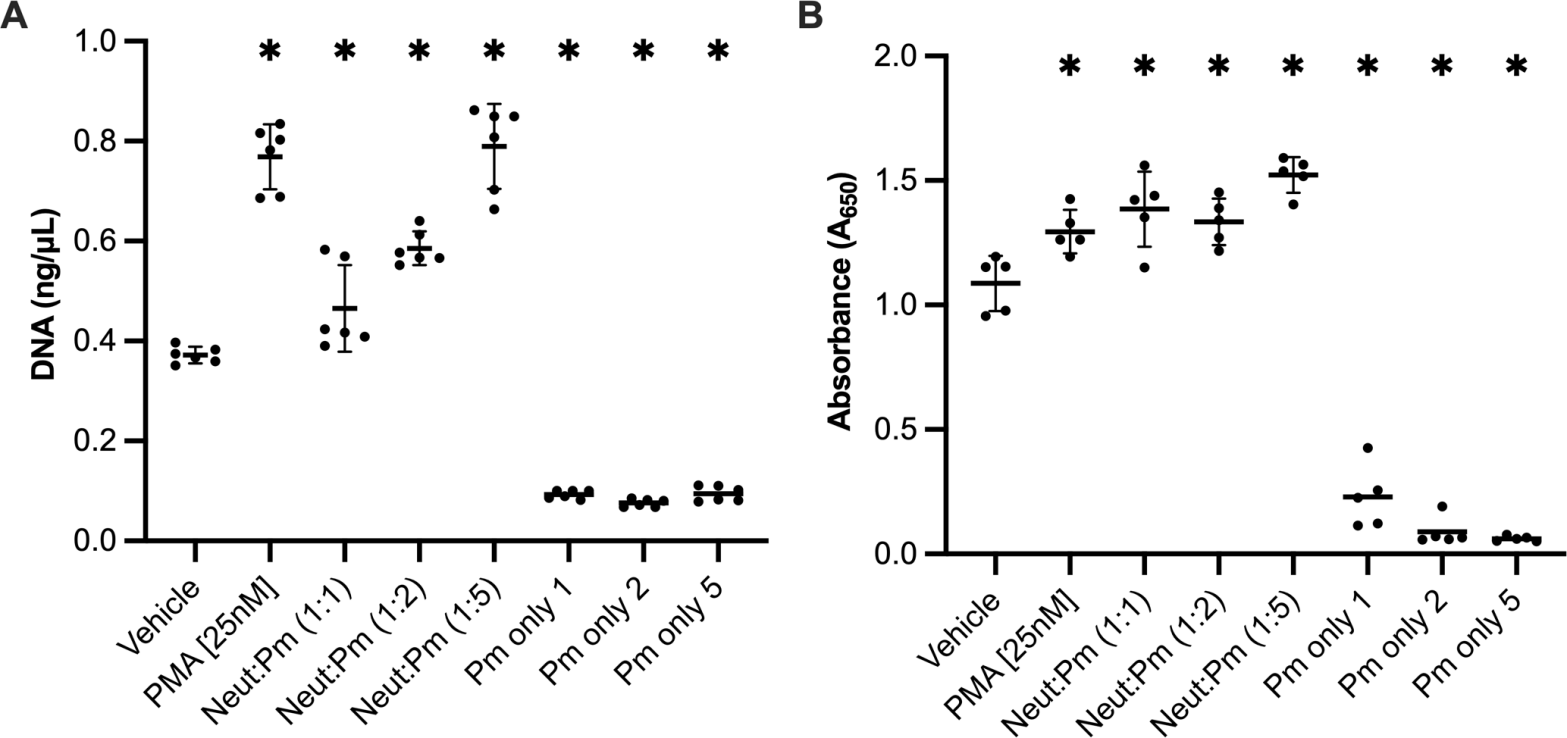
*Pnuemocystis murina* stimulates NET production in bone marrow-derived neutrophils. Bone marrow-derived neutrophils were stimulated with vehicle, phorbol myristate acetate (PMA; 25 nM; positive control), and *P. murina* at different multiplicities of infection (1, 2, or 5). Controls were established using the same concentrations of *P. murina* organisms without neutrophils.(**A**) Culture supernatant was assessed for extracellular DNA released from neutrophils. (**B**) Culture supernatant assessed for MPO-DNA complexes by ELISA. One-way ANOVA. *, *P* < 0.05 compared to vehicle.

Considering that *Pneumocystis* infections result in lung neutrophilia and NETs were observed within the lungs of *P. murina*-infected mice, we investigated whether *P. murina* could directly induce neutrophils to undergo NETosis and produce NETs. In experiments using bone marrow-derived neutrophils, we found that *P. murina* stimulated the release of NETs in neutrophils, as evidenced by the extracellular DNA released into the supernatant in a dose-dependent manner (Fig. 4A). Additionally, since NETs carry various proteins, we conducted a sandwich ELISA to detect MPO-DNA complexes within the NETs, which showed that *P. murina* stimulated the release of DNA-MPO complexes from neutrophils (Fig. 4B).

**Fig 5:**
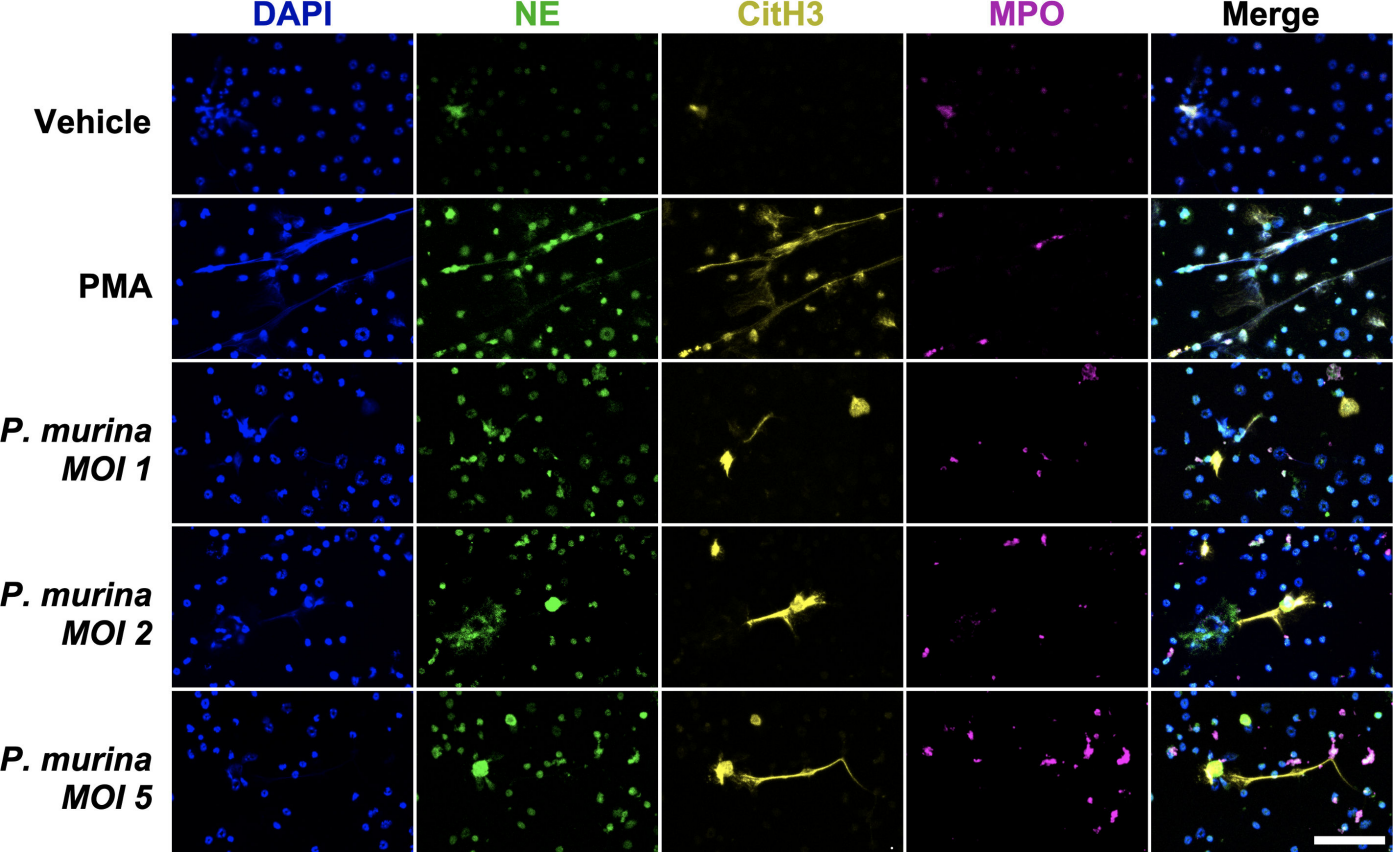
*Pneumocystis murina* directly stimulates NET production in bone marrow-derived neutrophils. Immunofluorescence of the bone marrow-derived neutrophils stimulated with vehicle, phorbol myristate acetate (PMA; 25 nM), or *P. murina*. The NET production was seen in PMA- and *P. murina*-treated neutrophils as shown by neutrophil elastase (green), citrullinated histone H3 (yellow), MPO (magenta) expression. DAPI, blue. Scale, 50 µm.

Immunofluorescence analysis showed the presence of NET structures, as seen by expelled DNA decorated with NE, CitH3, and MPO (Fig. 5). These data indicate that *P. murina* can directly stimulate the production of NETs *in vitro*.

### NETs are detrimental to *P. murina* viability

**Fig 6:**
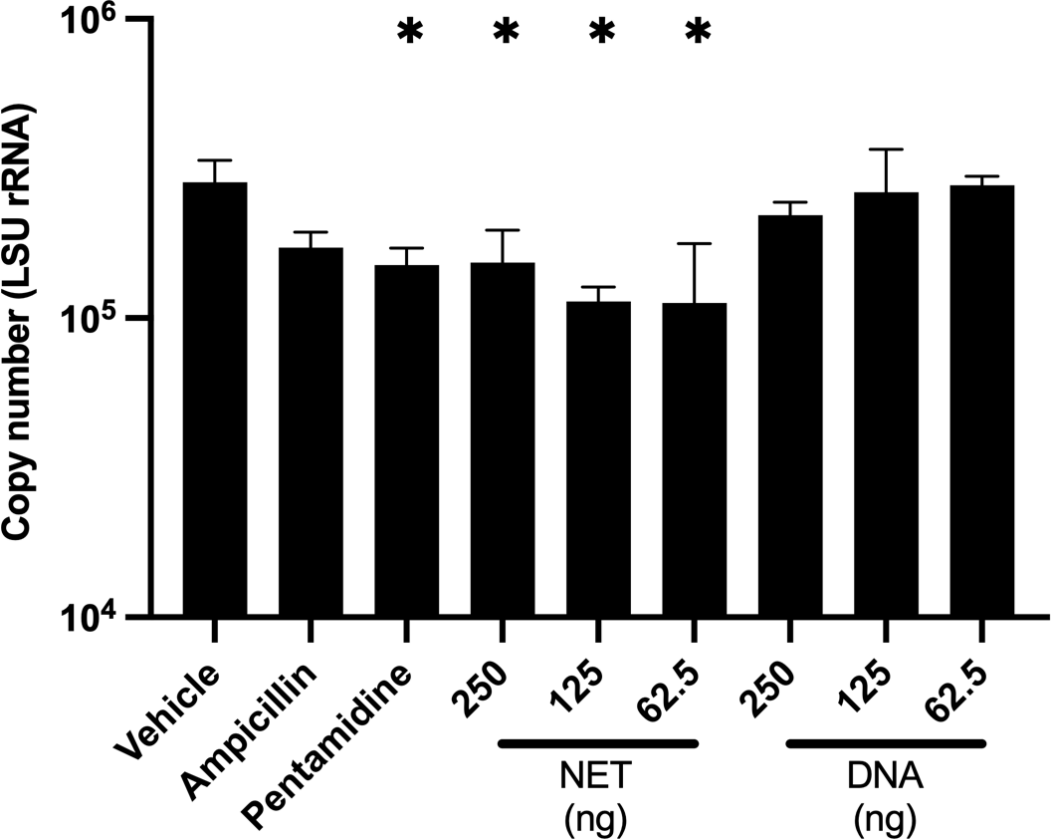
NETs are detrimental to *Pneumocystis murina* viability. *P. murina* (*n* = 3; 5 × 10^7^ nuclei) were inoculated into 96-well plates (Costar 3548, Corning, New York). Isolated NETs from PMA (25 nM)-stimulated neutrophils or salmon sperm DNA samples, used as control for non-complexed DNA, were added to the wells. Plates were incubated at 5% CO_2_, 37°C. After 24 hours, *P. murina* was quantified by large subunit (*LSU) rRNA* copy number by qPCR. One-way ANOVA. *, *P* < 0.05 compared to vehicle.

NETs are known to play a role in pathogen trapping and killing. Given that *P. murina* can directly stimulate the production of NETs in neutrophils *in vitro*, we investigated whether NETs were toxic to *P. murina*. After treating *P. murina* with NETs isolated from phorbol myristate acetate (PMA)-treated neutrophils, we observed decreased viability of *P. murina* (Fig. 6). These data indicate that NETs negatively impacted the viability of *P. murina*.

## DISCUSSION

*P. jirovecii* pneumonia remains a significant concern, particularly in patients with HIV/AIDS and those undergoing immunosuppressive treatments for conditions such as hematological cancers. Immunosuppressed hosts with PjP experience an influx of various immune cells, including T lymphocytes, polymorphonuclear neutrophils, and other leukocytes, resulting in profound inflammation and considerable morbidity and mortality. Traditionally, the focus of research on host responses to *Pneumocystis* has been centered around macrophage responses and role in pathogen clearance. However, our findings highlight the overlooked roles of neutrophils and the NLRP3 inflammasome in the host immune response during *P. murina* infection.

Neutrophils, despite their abundance in infected lungs, have been largely overlooked in the context of immune response to *Pneumocystis*. These cells participate in the inflammatory response during *P. murina* infection, as indicated by the increased expression of NLRP3 inflammasome-related genes observed in the lungs (Fig. 2). Moreover, our study identified NE and citrullinated histone H3, NET markers of NETosis, in the lungs of *P. murina*-infected mice (Fig. 3), indicating that this process was active during PmP. Our *in vitro* experiments further demonstrated that *P. murina* can directly stimulate NETosis in isolated neutrophils (Fig. 4 and 5).

Neutrophils are recognized as a critical line of defense against various pathogens. Although previous studies had questioned their significance in *Pneumocystis* infections and the clearance of *Pneumocystis*, our results indicate that neutrophils may contribute to the host’s inflammatory response by initiating NLRP3-inflammasome assembly and undergoing NETosis. The increased expression of NLRP3 inflammasome-related genes and the presence of NETs suggest that neutrophils are actively involved in the immune response against *P. murina*.

Importantly, uncontrolled inflammasome activation and NETs can drive excessive inflammation and tissue damage. Previous research has demonstrated that NETs can exacerbate lung injury and disrupt barrier function in mice, while disruption of NET formation or NET degradation led to decreased tissue damage and increased survival in mice (37–39). Our study highlights the importance of understanding the role of neutrophils and NET formation during *Pneumocystis* infection, as they may be driving increased inflammation, immune cell recruitment, and tissue damage.

Here, we demonstrated the NETs were detrimental to *P. murina* viability *in vitro* (Fig. 6). Similar deleterious impacts of NETs on the fungal viability are shown in *Candida albicans* (40), *Paracoccidioides brasiliensis* (41), and *Scedosporium apiospermum* (42). While NETs exhibit some control over *Aspergillus fumigatus* infection, they are insufficient for complete eradication of the fungus (43). Moreover, biofilms provide a protective extracellular matrix which can resist the killing effect of NETs (44). *C. albicans* form protective biofilms that inhibit NET formation and secrete nucleases to degrade NETs (45). *Pneumocystis* forms biofilms (46), which may confer protection against complete eradication of the fungi by NETs. *Pneumocystis* biofilms may be utilizing extracellular DNA as a scaffold to form a biofilm, similar to *C. albicans* and *A. fumigatus*, where DNA serves as a crucial component in biofilm matrices (47–49). Furthermore, the integrity of these biofilms can be disrupted by DNase, potentially increasing susceptibility to antifungal therapies (49, 50).

In conclusion, our research provides valuable insights into the role of neutrophils, particularly their involvement in NLRP3 inflammasome activation and NETosis, in the host immune response during *P. murina* pneumonia. By elucidating the participation of neutrophils and their ability to form NETs in response to *P. murina*, we expand our understanding of the intricate inflammatory dynamics at play during *Pneumocystis* infection. These findings offer potential avenues for therapeutic interventions aimed at modulating the immune response in individuals at risk of or affected by PjP. Further investigations are warranted to delve into the precise mechanisms underlying neutrophil-mediated inflammation, inflammasome activation, and NETosis in PjP as well as to explore the potential therapeutic strategies that may arise from these discoveries.

## MATERIALS AND METHODS

### Animals

Male C3H/HeNCrl (5 weeks old; Charles River, Raleigh, NC) mice were housed under barrier conditions with autoclaved food and bedding in sterilized cages equipped with sterile microfilter lids. Mice were immunosuppressed with dexamethasone (4 mg/L) in acidified drinking water, available *ad libitum*. The mice were infected by co-housing with *P. murina*-infected mice. Infection was allowed to progress for 5 weeks (to represent a moderate infection; 5wI) and 7 weeks (high infection; 7wI). Time-matched immunosuppressed, uninfected mice were used as controls (moderate and high control; 5wC and 7wC). Mice were euthanized humanely, and their lungs removed for quantification of fungal burdens (*n* = 3), or the lungs were flash frozen in liquid nitrogen, ground using a mortar and pestle, and then stored at −80°C for RNA sequencing (*n* = 5). To quantify fungal burden, lungs were homogenized in PBS using gentleMACS (Miltenyi Biotec, Auburn, CA, USA), then stained with a modified Diff-Quik staining (51) to visualize the nuclei for microscopic enumeration. The microscopic counts were log transformed, and values were compared by the one-way analysis of variance.

### RNA sequencing

The following steps were performed at UC Genomics, Epigenomics, and Sequencing Core, Department of Environmental Health, University of Cincinnati, Cincinnati, OH. RNA was extracted from lung tissue using mirVana miRNA Isolation Kit (Invitrogen, Carlsbad, CA, USA). The Ribo-Zero Gold (Human/Mouse/Rat) and (Yeast) kit (Illumina, San Diego, CA, USA) were used to deplete rRNA using 300 ng total RNA as input. The isolated RNA was RNase III fragmented and adaptor ligated using PrepX mRNA Library Kit (Takara, Mountain View, CA, USA), then converted into cDNA using Superscript III reverse transcriptase (Lifetech, Grand Island, NY, USA). Resulting cDNA was purification using Agencourt AMPure XP beads (Beckman Coulter, Indianapolis, IN, USA). Barcode index was added using a universal and index-specific primer with PCR to each ligated cDNA sample, and the amplified library was enriched by AMPure XP beads purification.

The quality and yield of the purified library were analyzed by Bioanalyzer (Agilent, Santa Clara, CA, USA) using DNA high-sensitivity chip. Libraries were quantified by qPCR measured by Kapa Library Quantification Kit (Kapa Biosystems, Woburn, MA, USA) using ABI’s 9700HT real-time PCR system (Lifetech, Grand Island, NY, USA). Libraries at the final concentration of 12.0 pM were clustered onto a flow cell using Illumina’s TruSeq SR Cluster Kit v3 and sequenced for 50 cycles using TruSeq SBS Kit on Illumina HiSeq system.

### RNAseq analysis

Raw reads were trimmed for quality and barcode removal using fastp (52). Trimmed reads were aligned to the *M. musculus* genome (accession GCF_000001635.27) and quantified using salmon (53). DEseq2, using an absolute fold change >1.5 and FDR-adjusted *P*-value of 0.05, was used to identify differentially expressed genes between groups (54). Ensemble of Gene Set Enrichment Analyses was performed to identify pathways with enriched gene expression with an absolute fold change >1.0 and FDR-adjusted *P*-value of 0.05 (55).

### ELISA

Protein was extracted from uninfected and *P. murina*-infected lung tissue (*n* = 3) using radio-immunoprecipitation assay buffer. Protein was quantified by BCA Protein Assay Kit (Thermo Scientific, Rockford, IL, USA) and normalized to 2 mg/mL. MPO and NE were quantified from the protein lysate using Mouse MPO ELISA Kit (Abcam ab155458, Cambridge, UK) and Mouse Neutrophil Elastase ELISA Kit (Abcam ab252356, Cambridge, UK) following manufacturer instructions.

### Immunostaining

Lung tissue (*n* = 3) was fixed in neutral buffered formalin for 24 hours at room temperature. The tissue was then dehydrated and stored in 70% ethanol. Fixed lungs were embedded with paraffin, then sliced at 10 um, and mounted on positive-charged slides. Slides were deparaffinized using a xylene series, followed by ethanol exchange, then rehydrated in water. Epitope retrieval was performed using citrate buffer at 91°C for 90 minutes. Anti-NE (1:500; Abcam ab68672, Cambridge, UK) or Anti-MPO (1:100; Abcam ab9535, Cambridge, UK) was incubated with the sections for 1 hour, washed, and then probed with anti-rabbit-HRP (1:1,000; Roche Diagnostics UltraMap 05269717001, Indianapolis, IN). Colorimetric signal was developed using diaminobenzidine and then counterstained with hematoxylin.

Neutrophils (4 × 10^4^; performed in triplicate) were grown on 18-mm glass coverslips (Electron Microscopy Sciences; Hatfield, PA, USA) in a 12-well dish (CytoOne, Ocala, FL, USA). After NETosis experiments, cells were fixed in 3.7% formaldehyde in PBS for 15 minutes and permeabilized with 0.1% Triton-X for 10 minutes.

Neutrophil samples were blocked in 10% goat serum for 1 hour, then incubated with anti-Neutrophil Elastase-AlexaFluor 488 (1:100; Santa Cruz Biotechnology, Dallas, TX), anti-Citrullinated Histone H3 (1:100; Abbomax, San Jose, California), or anti-Myeloperoxidase (1:100; Santa Cruz Biotechnology, Dallas, TX) in blocking buffer for 1 hour. Secondary goat anti-rabbit antibody conjugated with AlexaFluor 594 (1:1,000; Invitrogen, Carlsbad, CA) and goat anti-mouse IgG antibody conjugated with AlexaFluor 647 (1:1,000; Invitrogen, Carlsbad, CA) in blocking buffer were added and incubated for 2 hours in the dark. Cells were washed between incubations with 0.1% Tween 20 in PBS (PBST) three times for 15 minutes. DNA was stained with DAPI (1 ug/mL). Images taken on a Nikon A1 Confocal Microscope at Cincinnati Children’s Hospital Medical Center or on a Leica Stellaris confocal microscope at the University of Cincinnati Live Microscopy Core.

### Neutrophil isolation

Bone marrow was obtained from the humerus, femur, and tibia of immunocompetent C3H/HeNCrl male mice (*n* = 3 groups of pooled bone marrow from two mice). Red blood cells were lysed in 0.08% ammonium chloride for 10 minutes on wet ice and then washed with RPMI 1640 (Gibco, Grand Island, NY, USA) twice. Neutrophils were isolated by positive selection using Anti-Ly-6G MicroBeads UltraPure (Miltenyi Biotec, Auburn, CA, USA) and resuspended in RPMI 1640.

### NETosis assay

Neutrophils (1 × 10^6^) were plated onto a 24-well dish (CytoOne, Ocala, FL, USA). Cells were then treated with either RPMI 1640 vehicle, phorbol myristate acetate (25 nM in RPMI 1640; Millipore; Burlington, MA, USA), or *P. murina* at multiplicities of infection of 1, 2, or 5. Cells were incubated at 37°C 5% CO_2_ for 4 hours to allow NETosis to occur (56). Neutrophils were then gently washed twice with PBS. Cells were resuspended in 500 µL PBS by using a cell scraper to lift the neutrophils from the plate surface, then centrifuged at 300 × *g* to separate cell debris from NET material. These experiments were performed in triplicate using isolated neutrophils from three independent groups, as described above. The NET-containing supernatants were collected for downstream analysis.

### Quantification of extracellular DNA

Extracellular DNA release was quantified from the supernatant of NETosis-stimulated neutrophils using the QuantiFluor dsDNA system (Promega, Madison, WI) per manufacturer instructions. Briefly, samples and dsDNA standards were stained with QuantiFlour dsDye, and fluorescence (504nm_Ex_/531nm_Em_) was measured using a BioTek Synergy HTX plate reader.

### Detection of MPO-DNA complexes

NET release from the supernatant of NETosis-stimulated neutrophils was determined by detecting complexes of DNA and MPO. High-binding 96-well plates (Corning 2592, Kennebunk, ME, USA) were coated overnight with capture antibody, anti-MPO (1:500; Invitrogen; Carlsbad, CA, USA), in 50 mM carbonate buffer, pH 9.4 at 4°C. After that time, plates were washed three times with PBS with 0.1% Tween 20. Wells were blocked with StartingBlock PBS Blocking Buffer (Thermo Scientific, Rockford, IL, USA) for 2 hours at room temperature, then washed three times with PBST. Cell supernatant from each treatment was diluted 1:10, and 100 µL was added to the wells and incubated at 4°C for 24 hours, then washed three times in PBST. Detection antibody, anti-DNA-HRP (1:100; Zymo Research, Irvine, CA, USA), was added to the plate and incubated for 24 hours. After washing three times in PBST, colorimetric signal was detected using TMB (3,3',5,5' tetramethylbenzidine; Thermo Scientific, Rockford, IL, USA). Absorbance (650 nm) was measured using a BioTek Synergy HTX plate reader.

### NET toxicity

Isolated NETs from the supernatant of PMA-stimulated neutrophils were collected and quantified as shown above. Salmon sperm DNA was used as a control for non-complexed DNA. *P. murina* (5 × 10^7^ nuclei) were inoculated into 96-well plates (Costar 3548; Corning, NY, USA). PBS vehicle, DNA, or NET samples were added to the wells in triplicates. Plates were incubated at 5% CO_2_, at 37°C. After 24 hours, total RNA was extracted from the samples using TRIZOL reagent (Life Technologies Inc.; Rockville, MD, USA) and Direct-zol-96 MagBead RNA (Zymo Research; Irvine, CA, USA), then reverse transcribed into cDNA using SuperScript IV VILO (Invitrogen, Carlsbad, CA,USA). *P. murina* organisms were quantified by large subunit (LSU) *rRNA* copy number, as described previously (57). Briefly, quantitative PCR was performed using TaqMan Fast Advanced Master Mix (Applied Biosystems, Waltham, Massachusetts, USA) following manufacturer instructions. Primers: *LSU rRNA* (ATGAGGTGAAAAGTCGAAAGGG; TGATTGTCTCAGATGAAAAACCTCTT; 6FAM-AACAGCCCAGAATAATGAATAAAGTTCCTCAATTGTTAC-TAMRA).

## Data Availability

Fastq files have been deposited to NCBI Sequence Read Archive under BioProject Accession: PRJNA1076530.
